# A Five-Year Retrospective Study of Foot-and-Mouth Disease Outbreaks in Southern Africa, 2014 to 2018

**DOI:** 10.1155/2021/7438809

**Published:** 2021-12-31

**Authors:** Elliot Mpolokang Fana, Sununguko Wata Mpoloka, Melvin Leteane, LaToya Seoke, Kelebogile Masoba, Mokganedi Mokopasetso, Aobakwe Rapharing, Tshephang Kabelo, Patricia Made, Joseph Hyera

**Affiliations:** ^1^OIE Sub-Saharan Africa Regional Reference Laboratory for Foot-and-Mouth Disease (OIE-SSARRLFMD), Botswana Vaccine Institute, Private Bag 0031, Gaborone, Botswana; ^2^Department of Biological Sciences, Faculty of Science, University of Botswana, Private Bag 00704, Gaborone, Botswana; ^3^National Veterinary Laboratory, OIE-SSARRLFMD, Botswana Vaccine, Private Bag 0031, Gaborone, Botswana; ^4^Veterinary Department, Botswana Vaccine Institute, Private Bag 0031, Gaborone, Botswana

## Abstract

Foot-and-mouth disease (FMD) virus (FMDv), like other ribonucleic acid (RNA) genome viruses, has a tendency to mutate rapidly. As such, available vaccines may not confer enough cross-protection against incursion of new lineages and sublineages. This paper is a retrospective study to determine the topotypes/lineages that caused previous FMD outbreaks in 6 southern African countries and the efficacy of the current vaccines to protect cattle against them. A total of 453 bovine epithelial tissue samples from 33 FMD outbreaks that occurred in these countries from 2014 to 2018 were investigated for the presence of FMDv. The genetic diversity of the identified Southern African Type (SAT)-FMD viruses was determined by comparing sequences from outbreaks and historical prototype sequences. Of the 453 samples investigated, 176 were positive for four FMDv serotypes. Out of the 176 FMD positive cases there were 105 SAT2 samples, 32 SAT1 samples, 21 SAT3 samples, and 18 serotype O samples. Phylogenetic analysis grouped the SATs VP1 gene sequences into previously observed topotypes in southern Africa. SAT1 viruses were from topotypes I and III, SAT2 viruses belonged to topotypes I, II, III, and IV, and SAT3 viruses were of topotypes I and II. Vaccine matching studies on the field FMDv isolates produced *r*_1_-values greater than or equal to 0.3 for the three SAT serotypes. This suggests that there is no significant antigenic difference between current SAT FMD vaccine strains and the circulating SAT serotypes. Therefore, the vaccines are still fit-purpose for the control FMD in the region. The study did not identify incursion of any new lineages/topotypes of FMD into the sampled southern African countries.

## 1. Introduction

Foot-and-mouth disease (FMD) is a contagious viral disease clinically characterized by lesions in the mouth and feet of cloven-hoofed animals; the disease affects more than 70 animal species [1–4]. FMD has a detrimental effect on the agricultural economies of most countries worldwide, where cattle, sheep, goats, and pigs play a vital role to the gross domestic product [5–8]. These susceptible livestock are essential for food security, livelihoods, cultural identity, and social status of about 200 million small-scale farmers in Africa especially in sub-Saharan Africa [9, 10]. In Africa, direct economic losses associated with reduction of livestock products (meat and milk) due to FMD infection are estimated at US$2.3 billion per year [11, 12]. Indirect losses from the disease occur as a consequence of restrictions on domestic, regional, and international trade [13], and these losses can overshadow the direct economic losses.

The causative agent of FMD is an *Aphthovirus* in the family *Picornaviridae* and is classified into seven distinguishable serotypes and their respective topotypes [14, 15]. Each of the seven FMDv serotypes is immunologically distinct; antibodies raised against one serotype cannot confer protection against another FMDv serotype and sometimes even within the same serotype [16]. Vaccine matching using *in vitro* assays such as virus neutralization test (VNT) [17] and enzyme linked immunosorbent assays (ELISA) is used to ensure efficacy of the vaccine [4, 18, 19]. All FMD virus (FMDv) serotypes except Asia 1 and C serotypes are endemic in Africa; serotype C was last isolated worldwide in 2004 [20]. Among the five FMDv serotypes that occur in Africa, the three SATs are unique to Africa [21, 22]; however there have been reports of its incursion outside the continent in the past [23, 24]. In southern Africa, the SAT serotypes have been closely linked to the African buffalo (*Syncerus caffer*) which is attributable to the continued presence of these FMDv serotypes in the region [20, 22, 25–27].

Worldwide, FMD viruses are maintained in two continents, Asia and Africa; in South America all countries except Venezuela are free with vaccination [20]. The viruses within these continents have evolved to adapt to the places where they occur and this has led to existence of seven FMDv Pools. Each FMDv Pool has a minimum of three FMDv serotypes [19].

Historically, FMD has been a scourge in the southern African region. An overview of the disease in the region over a 50-year period from 1931 to 1990 recorded a total of 350 epizootic outbreaks in some countries of southern Africa [28]. During the period from 2000 to 2014, FMD outbreaks in cattle in southern African countries (Botswana, Malawi, Mozambique, Namibia, Zambia, and Zimbabwe) have been caused mainly by SAT2 followed by SAT1 [21]. Consequently, vaccination programmes in the region have been targeting protection against primarily the two SAT viruses, using a bivalent inactivated vaccine with aluminium hydroxide and saponin adjuvant. The vaccination programmes have however not been carried out regularly according to manufacturer's recommendations [29], and this leads to inefficient vaccinations. It has been shown in Europe that regular prophylactic vaccination can successfully eradicate the disease [30]. Rather than instituting regular prophylactic FMD vaccinations, most countries in southern Africa have been and are still reacting to outbreaks; this happens possibly due to lack of funds to follow prescribed vaccination schedules. Therefore, outbreaks occur when animal immunity levels have fallen below the threshold of protection [30]. Lack of regular postvaccination immunity assessments by respective Directorates of Veterinary Services in the region makes it impossible to identify antibody decay; thus vaccination schedules are erratic [31]. These challenges call for a new approach of controlling the disease in endemic settings such as southern Africa.

Most information about FMD outbreaks in southern Africa is outdated and underreported. In this publication, a retrospective study of FMD outbreaks which occurred between 2014 and 2018 in 6 southern African countries, namely, Botswana, Malawi, Mozambique, Namibia, Zambia, and Zimbabwe, is presented. This study aimed to update the currently available reports on FMD outbreaks in this region. Topotype identification and antigenic characterization for virus strain differentiation done in this study can also help facilitate selection of appropriate vaccines to control the disease outbreaks.

## 2. Materials and Methods

### 2.1. Sample Collection and Submission

Epithelial tissue samples were collected during active FMD outbreaks from cattle exhibiting clinical signs and lesions suggestive of infection with FMDv by veterinarians of the affected countries. Over the study period (2014–2018), a total of 453 cattle epithelial tissues were received from eleven southern African countries for confirmatory diagnosis of FMD at the OIE sub-Saharan African Regional Reference Laboratory for FMD (OIE-SSARRLFMD) based at Botswana Vaccine Institute (BVI). The samples were preserved in transport medium containing equal volumes of glycerol and phosphate-buffered saline at pH 7.2–7.6 with antibiotics [6, 32–34]. The samples were packaged and labelled properly in accordance with United Nations/International Air Transport Association (UN/IATA) dangerous goods standards and stored frozen until shipment [35]. The samples were shipped together with veterinary import permits from the Director of Veterinary Services (DVS) of Botswana and export veterinary permits from the DVS of the respective submitting countries and a detailed request form for test(s). Upon receipt, the samples were stored at −80°C until testing.

### 2.2. Sample Preparation for Virus Isolation and RNA Extraction

Bovine epithelial tissue samples were processed for virus isolation and extraction of FMDv ribonucleic acid (RNA) as described in the OIE Manual of Diagnostic Tests and Vaccines for Terrestrial Animals [36]. The procedure followed has been presented elsewhere [32, 34]. Briefly, the samples were thawed at room temperature and blot-dried on absorbent paper to remove the preservative (transport medium). Approximately 10% suspension of the tissue sample was made by mixing 1 gram of ground epithelial tissue with 9 ml tissue culture medium, Eagles Minimum Essential Medium (10% Minimum Essential Medium (MEM 10×), 10% lactalbumin hydrolysate, 4.5% sodium bicarbonate, 1% calf serum, 0.2% penicillin, and sterile distilled water at pH 7.4). After clarification at 2000 g for 10 minutes at 4°C, the suspension was divided into two equal parts; one portion was used for virus isolation and the other was used for RNA extraction and reverse-transcription polymerase chain reaction (RT-PCR) to amplify the VP1 region for sequencing.

### 2.3. Virus Isolation and Typing

The procedure for FMDV isolation and typing as used by the RRLSSA-FMD has been well documented [34]. Virus isolation is done in primary lamb kidney cell monolayers grown in 25 cm^3^ Corning® cell culture flasks from Sigma Aldrich. Each flask was inoculated with 500 *μ*l epithelial tissue suspension and thereafter incubated at 37°C for 1 hr in a Thermo Fisher Scientific CO_2_ incubator to allow the FMDv if any in the sample to infect the cells. Then cell culture medium (15 ml) was added to each cell culture and further incubated at 37°C for 48 hrs. FMD virus infected cells undergo degenerative changes, technically termed cytopathic effect (CPE). Such changes usually appear after 24 hrs but with very virulent virus strains they may appear as early as 12 hrs. After inoculation and incubation at 37°C, the cultures were examined microscopically daily using inverted light microscope (Olympus CKX31, Olympus Corporation, Tokyo, Japan) for evidence of CPE. If no CPE was observed after 48 hrs, the cultures were frozen at −70°C and then processed for second passage as described elsewhere [34]. If still no CPE was observed on second passage, the latter was repeated for a third and last time (third passage). Test samples including positive and negative control samples were considered negative for VI if no CPE was observed after three passages of the sample and the cultures were discarded following biosecurity procedures. Aliquots of cell culture fluids in cell cultures exhibiting CPE (positive for FMDV) were further tested by antigen (Ag) ELISA for FMDv serotypes O, A, SAT1, SAT2, and SAT3 in order to identify the serotype involved in the outbreak. The Ag-ELISA was performed as described in the OIE Terrestrial Manual [36].

### 2.4. RNA Extraction and RT-PCR

Total RNA was extracted from 200 *μ*l of supernatant obtained from clarification of crushed clinical epithelial tissues. The ZR-Viral RNA kit™ (Zymo Research Corporation, California, USA) was used for total RNA extraction following the user manual instruction with minor changes, the start volume of 200 *μ*l instead of 100 *μ*l, and 15 *μ*l elution buffer instead of 50 *μ*l [37].

Using prior knowledge of the sample serotypes from antigen ELISA, serotype specific primer pairs were used. The forward primers were different for each serotype and these were SAT1U-OS, SAT2-P1-1223F, and SAT3-1C559F. The reverse primer SAT 2B208R was used for SAT1, 2, and 3 as described by Knowles and colleagues [38]. The primer pairs were used to amplify the highly variable FMD virus VP1 gene using conventional RT-PCR. The OneTaq One-Step RT-PCR kit from New England Biolab (NEB) was used according to the manufacturer's recommended protocol. The master mix without the RNA template was prepared in a clean room to avoid contamination as follows: OneTaq One-Step Reaction Mix (2X) 25 *μ*l, OneTaq One-Step Enzyme Mix (25X) 2 *μ*l, Gene-Specific Forward Primer (10 *μ*M) 2 *μ*l, Gene-Specific Reverse Primer (10 *μ*M) 2 *μ*l, and total RNA (1 *μ*g) X*μ*l added in the RNA extraction room. RNA concentration was determined using the Qubit® 3.0 fluorimeter from Thermo Fisher Scientific. The final volume was made up to 50 *μ*l using nuclease-free water. The Applied Biosystems® Veriti® 96-well thermal cycler (Thermo Fisher Scientific, Waltham, MA USA) was used for RT-PCR using the following parameters: reverse-transcription (RT) at 48°C for 30 min/1 cycle; followed by initial denaturation at 94°C for 1 min/1 cycle, denaturation at 94°C for 15 s/40 cycles, annealing at 50°C for 30 s/40 cycles, and extension at 68°C for 1 min/40 cycles; and a final extension at 68°C for 5 min/1 cycle, and held at 4°C before agarose gel electrophoresis. A volume of 25 *μ*l of PCR product was mixed with 10 *μ*l gel loading dye (6X) from NEB and loaded onto respective wells on a 1.5% agarose gel stained with GelRed^TM^ (Biotium, Inc., Fremont, CA 94538, USA). The amplicon bands were visualized under UV light and correct size was determined from alignment with a 100 bp DNA molecular weight marker. The expected band sizes for primer pairs used were 1045 bp, 1279 bp, and 1034 bp for SAT1, SAT2, and SAT3, respectively, [38]. The DNA bands of interest were excised from the gel and purified using the Zymoclean™ Gel DNA Recovery Kit (Zymo Research Corporation, California, USA).

### 2.5. Sequencing of VP1

Sequencing of the VP1 gene was performed according to a procedure already described elsewhere [34, 38]. Briefly, both reverse and forward strands of the purified amplicons were sequenced using BigDye® Terminator v3.1 cycle sequencing kit (Applied Biosystems, California, USA). The decision tree by Knowles and colleagues [38] was used for selecting the most appropriate primer for sequencing the amplicons. For SAT1 and SAT3, the same PCR forward primers were used and SAT2-D was used as forward sequencing for SAT2 amplicon, and the same NK72 primer was used for all the SATs. The master mix was prepared in a no-template clean room to minimize contamination as follows: To get final volume of 20 *μ*l, 2 *μ*l of 5X sequencing buffer was mixed with 4 *μ*l BigDye® Terminator v3.1 Ready Reaction Mix and 1 *μ*l of appropriate sequencing primer at a concentration of 10 *μ*mol and target template (RT-PCR products of VP1 specific reactions) diluted to contain 5–20 ng complementary DNA (cDNA). The following sequencing thermocycling parameters were set in the Applied Biosystems® Veriti® 96-Well Thermal Cycler (Thermo Fisher Scientific, Waltham, MA USA), with a heated lid at 105°C to curtail evaporation: 96°C for 1 min and 25 cycles of 96°C for 10 s, 50°C for 5 s and 60°C for 4 min. The sequence product was purified using BigDye XTerminator™ Purification Kit (Applied Biosystems, California, USA) following the manufacturers protocol, and for a 20 *μ*l reaction volume the procedure was as follows: pulse-centrifuge the reaction plate, and then pipette 90 *μ*l of Sam™ Solution into each plate well, followed by 20 *μ*l of homogenised XTerminator™ solution. The plates were then sealed using a clear adhesive film and vortexed for 30 minutes after which the plate was pulse-centrifuged. The sequenced product was run on an Applied Biosystems® 3130xl Genetic Analyzer (Applied Biosystems, California, USA) using recommended run module as per instruction manual.

### 2.6. Phylogenetic Analysis

Phylogenetic analysis was carried out as described by Knowles and colleagues [38] with some minor modifications. Representative prototype FMDV sequences for each topotype, SAT1 (*n* = 17), SAT2 (*n* = 36) and SAT3 (*n* = 14), were obtained from the National Centre for Biotechnology Information (NCBI) database. VP1 sequences from representative outbreak samples received during the study period and vaccine strains were SAT1 *n* = 5, SAT2 *n* = 19, and SAT3 *n* = 5. These were combined with the prototype FMDV sequence to determine the ancestry of the outbreak strains. Multiple sequence alignment of the sequences was done using Clustal W [39] imbedded in MEGA version *X* [40]. The aligned sequences were used to estimate distance matrices using the Kimura 2-parameter nucleotide substitution model [41], in MEGA software version *X*. The evolutionary history was inferred by using the Maximum Likelihood method and Tamura-Nei model [42]. A 1000 bootstrap replicate option in the program was used to verify the tree topology while other parameters were left at default.

### 2.7. Vaccine Matching

The antigenic relationship of the field virus isolates to the vaccine strains was determined using the two-dimensional virus neutralization test (2dmVNT). The 2dmVNT was carried out using pools of bovine vaccinal serum and 100 tissue culture infecting dose (100 TCID_50_) of the homologous vaccine virus strain and the same dose of the field virus isolate [19].

The antigenic similarity which symbolises the antigenic match between the homologous vaccine virus and the heterologous field virus isolate expressed in relationship coefficients (*r*_1_-values) was calculated as described elsewhere [17] using the following formula:

(1) $$\begin{array}{c} r1=\frac{\text{reciprocal of titre of reference serum against field virus isolate}}{\text{reciprocal of titre of reference serum against vaccine virus strain}}. \end{array}$$

*r*_1_-values were interpreted as follows:

- *r*_1_-values greater than or equal to (≥) 0.3 indicate that there are no significant antigenic differences between the field and vaccine virus. A potent vaccine containing the vaccine strain was likely to confer protection.
- *r*_1_-values less than (<) 0.3 indicate that there are significant antigenic differences between the field and vaccine virus. A high potent vaccine can sometimes still confer protection, but not always.
- *r*_1_-values of zero (0) are given when no neutralization of the field virus isolate was observed at 100 TCID50.

## 3. Results

### 3.1. Foot-and-Mouth Disease Outbreaks in Southern Africa, 2014–2018

A total of 33 FMD outbreaks in cattle were reported during 2014–2018 period in southern Africa.  Table 1 shows the distribution of the outbreaks by country per year. The highest frequency of occurrence of the disease at 41.4% was observed in 2015 followed by an occurrence frequency of 20.7% in 2014 and 2018. In 2017 the disease frequency was at 10.3% while the lowest frequency of occurrence of the disease (3.4%) was recorded in 2016.

### 3.2. Virus Isolation and Serotype Identification over Five-Year Period 2014–2018

A total of 176 out of 453 bovine epithelial tissue suspensions (38.8%) cultured in primary lamb kidney cells produced cytopathic effect (CPE) suggestive of FMDv replication in the cells. The CPE was confirmed to be that of FMDv by antigen ELISA test using serotype specific rabbit antiserum.

### 3.3. Distribution of FMD Positive Samples by Virus Serotype in Southern Africa, 2014–2018

**Figure 1:**
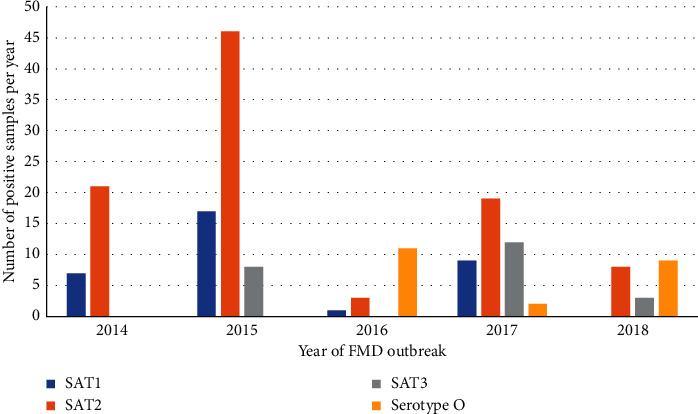
Annual distribution of foot-and-mouth disease (FMD) positive bovine samples by FMDv serotype observed in southern Africa, 2014–2018.

Out of the 176 positive samples, 32 were FMDv serotype SAT1, 106 were FMDv serotype SAT2, 21 were FMDv serotype SAT3, and 17 were FMDv serotype O. Overall, 159/176 of the outbreaks were caused by the SAT serotypes. The annual distribution of the FMD positive samples by serotype is shown in Figure 1.

**Figure 2:**
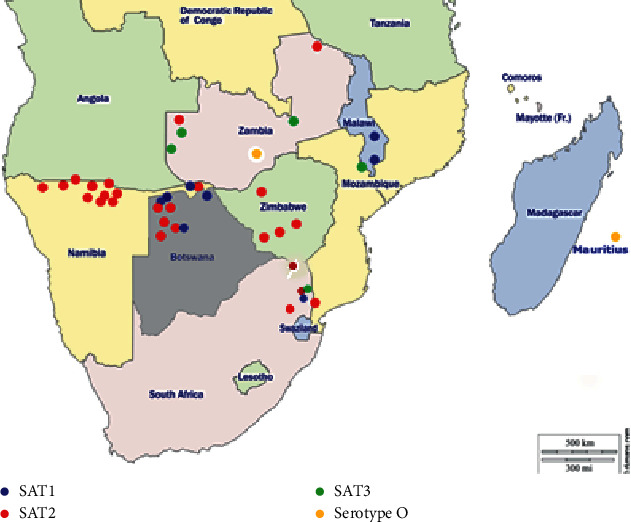
Map of southern Africa depicting FMD distribution between 2014 and 2018, by serotype (adapted with permission from http://www.d-maps.com).

Figure 2 shows the geographical distribution of FMDv serotypes isolated from cattle originating from various geographical locations in southern Africa. FMD virus serotype SAT2 was widely distributed, occurring in five countries of the region; FMD virus serotype SAT1 occurred in three countries and serotypes SAT3 and O were each detected in two countries of the region (Figure 2).

### 3.4. Genotyping of Virus Sequences of FMD Virus Serotypes Isolated, 2014–2018

**Figure 3:**
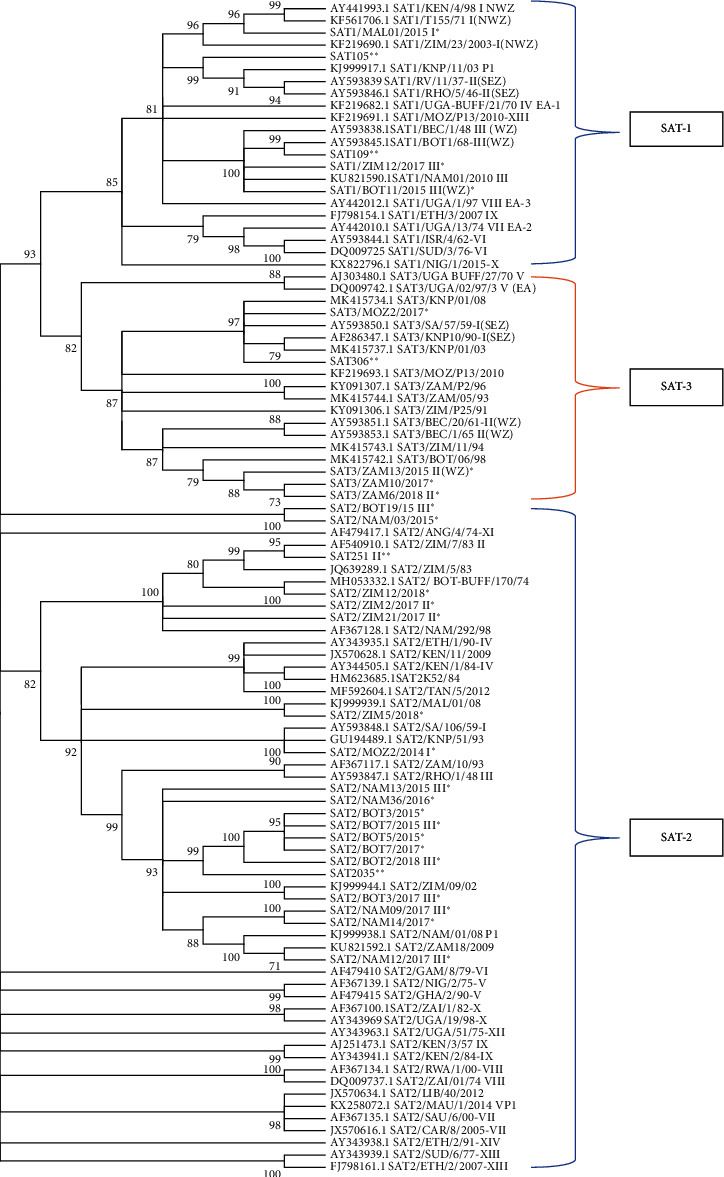
Evolutionary relationships of SATs strains and their topotypes. ^*∗*^ represents outbreak samples and ^*∗∗*^ are vaccine strains.

Genotyping of the FMDV VP1 sequences generated clustered the outbreak sequences with FMDs with their respective SAT serotypes. The outbreak FMD viruses represented SATs topotypes that had been isolated previously from the southern African region as shown in Figure 3.

### 3.5. Phylogenetic Analysis

The evolutionary history was inferred by using the Maximum Likelihood method and Tamura-Nei model [42]. The bootstrap consensus tree inferred from 1000 replicates is taken to represent the evolutionary history of the taxa analyzed. Branches corresponding to partitions reproduced in less than 70% bootstrap replicates are collapsed. The percentage of replicate trees in which the associated taxa clustered together in the bootstrap test (1000 replicates) is shown next to the branches. Initial tree(s) for the heuristic search were obtained automatically by applying Neighbour-Join and Bio NJ algorithms to a matrix of pairwise distances estimated using the Tamura-Nei model and then selecting the topology with superior log likelihood value. This analysis involved 96 nucleotide sequences. There were a total of 702 positions in the final dataset. Evolutionary analyses were conducted in MEGA *X*.

The phylogenetic analysis results shown in Figure 3 depict the evolutionary relationship of the outbreak strains with FMD viruses known to circulate in Africa. SAT1 FMD outbreaks were caused by viruses from topotypes I (NWZ) and III (NZ); SAT2 outbreaks were more diverse, covering topotypes I, II, III, and IV. Lastly SAT3 outbreaks were due to two topotypes I (SEZ) and II (WZ).

### 3.6. Vaccine Matching

Antigenic strain identification by vaccine matching using the two-dimensional virus neutralization test (2dmVNT) gave relationship coefficients (*r*_1_-values) of ≥0.3 indicating that the vaccine viruses were most likely going to confer protection against the heterologous field virus strains. Representative *r*_1_-values of the field virus strains analyzed in this study are shown in Table 2.

## 4. Discussion

FMD outbreaks are sporadically reported in most countries of the southern Africa region [27, 43, 44] and in the past decade (2004–2013), they have occurred in various countries of southern Africa [26]. The underreporting of FMD in the region contributes to sampling bias; only those serotypes that occur where reporting is done will seem prominent in the region. This paper is a retrospective review of FMD outbreaks to determine topotype/lineage that caused foot-and-mouth disease outbreaks in some southern African countries during the period between 2014 and 2018.

In this review, a total of 33 FMD outbreaks were sampled from seven southern African countries, Botswana, Malawi, Mauritius, Mozambique, Namibia, Zambia, and Zimbabwe. The causative viruses have been identified and characterized. Approximately 39% of the epithelial tissue samples (*n* = 453) collected from cattle exhibiting clinical FMD have demonstrated the presence of FMD virus (FMDv). During the period examined, a single outbreak was confirmed in a country hitherto free from FMD. This outbreak in cattle occurred in August 2016 in the Island of Mauritius; the disease outbreak was confirmed to be caused by FMDv serotype O which belonged to the Middle East South Asia (ME-SA) topotype; the disease was effectively controlled by vaccination [45]. The disease incursion into this country demonstrates that no country is safe from FMDv and therefore, the need for high surveillance and emergency preparedness, such as maintenance of strategic FMD vaccine banks, cannot be overemphasised [46].

Out of these outbreaks (*n* = 33), 30 out of 33 outbreaks were caused by FMD viruses in Pool 6 (SAT1, SAT2, and SAT3) while 3 out of 33 outbreaks were caused by FMD viruses in Pool 4. Of the outbreaks caused by Pool 6 virus serotype SAT2 contributed 20 of the 30 reported outbreaks, SAT1 contributed 6 out of 30 reported outbreaks, and SAT3 was the least reported, contributing 4 out of 30 outbreaks. Sporadic reporting and limited sampling during outbreaks might have supported the problem of sampling bias, which leads to virulent strains with higher propensity for transmission being sampled more. This sampling bias might lead to the wrong perception that the sampled strains are the predominant ones in the field. Sampling bias could be solved by intermittent sampling during periods of low incidence [47].

Outbreaks of FMD caused by FMDv serotype SAT3 have not been experienced in southern Africa for years until the recent emergence of the serotype in Zambia and Mozambique. Epidemiological intelligence seems to suggest that the outbreak in Zambia started in Shangombo district situated in the western province of the country and clinical cases in cattle were first reported in October 2015; the cases then spread to the neighbouring districts of Kalabo and Sikongo; further spread of the disease was controlled by a ring vaccination of 109,211 herd of cattle [48]. The primary affected district of Shangombo shares a border with Angola. Reflecting on the topotype of the virus isolate, topotype II (WZ), it is possible that the disease might have originated from transboundary movement of infected cattle between neighbouring countries without efficient animal movement control measures and synchronized disease surveillance programmes [6, 22, 49, 50]. Due to minimal surveillance occurring among these neighbouring countries, very little is known about the true incidence of FMD in both countries, and no official information is available on the isolation of FMDv serotype SAT3 from Angola. An outbreak of FMD was recorded in 2009; however, no virus could be isolated [26]. Absence of FMD epidemiological data in this country presents a challenge with regard to effective control of FMD in southern Africa as such information is important for effective vaccination [15].

Control strategies using a combination of methods such as physical separation of wildlife and livestock using game-proof fences, repeated vaccination of cattle herds exposed to wildlife, control of livestock movements, and careful assessment of risk of FMDv introduction into FMD-free areas are essential to ensure effective control and prevention of FMD [7, 44, 49]. These control strategies have been used in southern Africa and have greatly reduced the prevalence of the disease in the region albeit at a huge cost. FMD control by vaccination in southern Africa involves use of inactivated whole particle FMD vaccine adjuvanted with aluminium hydroxide gel and saponin. Some producers of these vaccines claim a homologous potency of 3PD50/dose. There is, however, no information on potency of African FMD vaccines in the public domain, and this limits discussions on efficacy of these vaccines. To implement effective control measures against FMD, certain countries in the region, for example, Botswana and Zimbabwe, have been demarcated into disease zones by codon fences [51]. Areas away from game conservancies have been classified as “green zones” and livestock in these zones are FMD-free without vaccination. Areas close to game conservancies are classified as “red zones” and are considered potentially infectious for FMD. Cattle in these zones are regularly vaccinated with trivalent vaccine containing SAT1, 2, and 3 strains. Strategically, most countries in southern Africa have been using largely bivalent FMD vaccine (SAT1 and SAT2). Only Botswana and Republic of South Africa have been using trivalent FMD vaccine (SAT1, SAT2, and SAT3). These vaccines are supplied as either semipurified vaccines or highly purified vaccines; the latter vaccines allow the differentiation between infection and vaccine induced antibodies, the so-called DIVA or marker vaccines.

The vaccine matching studies conducted during this period (2014–2018) have given *r*_1_-values greater than or equal to 0.3 (Table 2). These results show that the vaccines are capable of protecting against field challenge by FMD viruses [17]. These results are in agreement with the phylogenetic analysis results (Figure 3). The VP1 sequences from outbreaks strains were clustered in their respective serotypes, together with some vaccine strains used in the region, demonstrating a close evolutionary relationship. Some of the outbreaks clustered with sequences obtained from buffalo; this demonstrates the contribution this species has in the maintenance of FMD outbreaks in the region, and the results agree with previous studies conducted in the region [52]. The clustering together in phylogenetic trees (Figure 3) demonstrates a closeness in ancestry. However, extrapolation between nucleotide homology and antigenicity needs to be made with due care as there is not enough information available on the effect of amino acid changes on antigenicity [53].

The resurgence of FMDV serotypes SAT3 and O in southern Africa resulted in some countries revisiting their vaccination strategies; Zambia now has to vaccinate against all the three serotypes in Pool 6 as well as against serotype O from Pool 4. Mozambique and Namibia now also use a trivalent vaccine (SAT1, SAT2, and SAT3) instead of the bivalent vaccine (SAT1 and SAT2) for the control of FMD. The burden of FMD control by vaccination in Zambia has therefore increased and they might not be able to prevent the spread of the exotic viruses (Pool 4) further south to Zimbabwe, Botswana, and Namibia. It is henceforth suggested that concerted efforts coordinated by SADC and backed by international donor agencies such as the FAO, World Bank, and/or the EU be launched as a matter or emergency to provide effective control and prevention of FMD associated with exotic viruses spreading into the region from eastern Africa.

Elsewhere on the African continent, the control of FMD by vaccination is not regularly conducted. Many countries including those in southern Africa continue to vaccinate cattle in response to outbreaks rather than conducting regular prophylactic vaccinations of at least twice a year as the antibody decay after vaccination is approximately six months [54, 55]. Most countries on the continent are of low income, lack good veterinary infrastructures, lack skilled human resources, and are unable to control livestock movements. Therefore, they cannot afford the current conventional FMD vaccines. These constraints have rendered many countries in Africa exposed to the spread of FMD [12, 56, 57]. Notwithstanding these constraints, it is therefore imperative that countries in southern Africa and elsewhere on the African continent adhere to the recommendations of prophylactic vaccination of important susceptible hosts, particularly cattle, in order to get the best results with these vaccines. Going forward, developments of more cost-effective FMD vaccines rather than the current vaccines in use needs to be looked into; such vaccines might encourage more countries in the region and in Africa in general to institute regular prophylactic vaccination regimes to control and prevent the scourge of FMD. These regular vaccination programmes would prevent outbreaks of the disease in cattle and other susceptible livestock species and therefore facilitate regional and international trade of livestock and livestock products which would eventually lead to poverty eradication, prosperity, and food security.

The epidemiology of FMD in the African continent is complex and poorly understood; this constrains the ability to implement control strategies suitable for the continent [27]. As such, this presents a hindrance to full implementation of the global FMD control strategy developed by FAO and EuFMD and endorsed by the OIE, a strategy commonly referred to as the Progressive Control Pathway for Food-and-Mouth Disease (PCP-FMD) [28]. The PCP-FMD is a risk and evidence-based framework to guide endemic countries to progressively improve the management of FMD risks and reduce disease impacts and viral circulation [26]. This study identified strains present in the region, thus contributing towards one of the strategies within the PCP-FMD framework.

A lot is known about the molecular epidemiology of FMD caused by SAT serotypes of FMDv in southern Africa [22, 27, 52]. The findings of the current study contribute more information to the past knowledge regarding the molecular epidemiology of FMD in southern Africa. The SAT1 outbreaks were caused by viruses from topotype I (NWZ) and topotype III (WZ); the SAT2 viruses were the most prevalent and were caused by viruses from topotypes I to IV. Finally, SAT 3 outbreaks were sporadic; the main topotypes involved in the outbreaks were topotype I and II. The FMDv capsid protein, VP1, is the most heterogeneous protein among FMDv strains. Differentiation between FMDv strains can be obtained from VP1 nucleotide differences; a 15% nucleotide difference is the cut-off among serotypes O, A, C, and Asia 1 [4] while a 20% difference is used to differentiate strains among the SATs, because of their higher genetic variability when compared to the other serotypes [27]. Topotypes are immunologically different from one another and may require specific vaccines to ensure efficient control [4, 21, 22]. The immunological diversity observed among FMDv serotypes and strains together with uncontrolled animal movement in most parts of Africa contributes to difficulty in FMD control [21, 22, 26]. This data is vital for the development of topotype-specific tailored second generation FMD vaccines which might be more cost-effective than the FMD vaccines currently being used in southern Africa and elsewhere on the African continent.

## 5. Conclusion and Recommendation

Foot-and-mouth disease continues to be a big constraint towards international trade of cloven-hoofed livestock for most countries in southern Africa and elsewhere where the international trade of cattle (beef) forms an integral part of the country's gross domestic product (GDP). In southern Africa, the main FMD virus strains circulating in the field in the order of dominance are SAT2 and SAT1; however SAT3 now constitutes a potentially big threat to Zambia and the countries that it closely borders with. The same trend in FMD distribution patterns was observed in 1995 by Thomson [28].

It is recommended that in those countries where FMD control by vaccination is practiced, especially in areas where cattle, sheep, and goats share the same habitat with the African buffalo (*Syncerus caffer*), regular vaccination of cattle using trivalent FMD vaccine (SAT1, SAT2, and SAT3 combination) be adopted to prevent the spread of these viruses to susceptible populations. In those countries where exotic FMDv serotypes have entered from countries of different FMDv Pools, the exotic serotypes should be included in the vaccination regimes. Countries in southern Africa and elsewhere in sub-Saharan Africa need to regularly submit FMD samples to OIE reference laboratories in order to monitor the FMD situation in the region and study any epidemiological shifts and antigenic variability of the circulating strains relative to the homologous strains used in vaccine manufacturing.

## Figures and Tables

**Table 1:** Distribution of foot-and-mouth disease outbreaks in cattle by country in some southern African countries, 2014–2018.

Country	Number of outbreaks (cases) per year					Total	RF ^*∗*^ (%)
	2014	2015	2016	2017	2018		
Botswana	2	4	0	0	2	8	24.2
Malawi	0	0	1	0	1	2	6.1
Mauritius	0	0	1	0	0	1	3
Mozambique	1	1	0	1	0	3	9.1
Namibia	1	4	0	1	0	6	18.2
Zambia	0	2	0	2	3	7	21.2
Zimbabwe	2	1	1	0	2	6	18.2
Total	6	12	2	4	8	**33**	
RF^*∗*^ (%)	18.8	37.5	6.3	12.5	25		

**Table 2:** Vaccine matching results on some field FMD viruses isolated from cattle originating from various countries of southern Africa during the period 2014–2018.

Field virus isolate	*r*_1_-values per vaccine virus strain^1^		
	SAT105	SAT251	SAT306
SAT1/BOT/05/2014	0.6	—	—
SAT1/BOT/17/2014	0.5	—	—
SAT2/MOZ/04/2014	—	0.7	—
SAT2/ZIM/03/2014	—	0.4	—
SAT2/ZIM/04/2014	—	0.3	—
SAT2/ZIM/05/2014	—	0.3	—
SAT2/ZIM/07/2014	—	0.4	—
SAT2/ZIM/09/2014	—	0.4	—
SAT1/BOT/11/2015	0.9	—	—
SAT2/BOT/15/2015	—	0.5	—
SAT2/BOT/21/2015	—	0.7	—
SAT2/NAM/09/2015	—	0.6	—
SAT2/NAM/20/2015	—	0.5	—
SAT1/NAM/42/2015	0.4	—	—
SAT3/ZAM/08/2015	—	—	0.4
SAT3/ZAM/09/2015	—	—	0.4
SAT2/ZIM/17/2015	—	0.8	—
SAT1/MAL/01/2016	0.4	—	—
SAT2/BOT/02/2018	—	0.6	—
SAT2/ZIM/02/2018	—	0.7	—
SAT3/ZAM/06/2018	—	—	0.7

*r*_1_-values greater than or equal to (≥) 0.3 indicate that there are no significant antigenic differences between the field and vaccine virus. BOT = Botswana, MAL = Malawi, MOZ = Mozambique, NAM = Namibia, ZAM = Zambia, ZIM = Zimbabwe.

## Data Availability

Data used in this article are included within the article.

## References

[1] Alexandersen S., Zhang Z., Donaldson A. I., Garland A. J. M. (2003). The pathogenesis and diagnosis of foot-and-mouth disease. *Journal of Comparative Pathology*.

[2] Bronsvoort B. M. D. C., Nfon C., Hamman S. M., Tanya V. N., Kitching R. P., Morgan K. L. (2004). Risk factors for herdsman-reported foot-and-mouth disease in the Adamawa Province of Cameroon. *Preventive Veterinary Medicine*.

[3] Carrillo C. (2012). Foot and mouth disease virus. *Viral Genomes - Molecular Structure, Diversity, Gene Expression Mechanisms and Host-Virus Interactions*.

[4] Samuel A. R., Knowles N. J. (2001). Foot-and-mouth disease type O viruses exhibit genetically and geographically distinct evolutionary lineages (topotypes). *Journal of General Virology*.

[5] Kitching R. P., Hughes G. J. (2002). Clinical variation in foot and mouth disease: sheep and goats. *Revue Scientifique et Technique de l’OIE*.

[6] Sinkala Y., Simuunza M., Pfeiffer D. U. (2014). Challenges and economic implications in the control of foot and mouth disease in sub-Saharan Africa: lessons from the Zambian experience. *Veterinary Medicine International*.

[7] Thomson G. R., Vosloo W., Bastos A. D. S. (2003). Foot and mouth disease in wildlife. *Virus Research*.

[8] Rodriguez L. L., Grubman M. J. (2009). Foot and mouth disease virus vaccines. *Vaccine*.

[9] Le Gall F., Leboucq N. (2004). The role of animal disease control in poverty reduction, food safety, market access and food security in Africa-Recueil des thèmes techniques présentés au Comité international ou aux Commissions régionales. *Control*.

[10] Perry B., Grace D. (2009). The impacts of livestock diseases and their control on growth and development processes that are pro-poor. *Philosophical Transactions of the Royal Society B: Biological Sciences*.

[11] Casey-Bryars M., Reeve R., Bastola U. (2018). Waves of endemic foot-and-mouth disease in eastern Africa suggest feasibility of proactive vaccination approaches. *Nature Ecology & Evolution*.

[12] Knight-Jones T. J. D., Rushton J. (2013). The economic impacts of foot and mouth disease - what are they, how big are they and where do they occur?. *Preventive Veterinary Medicine*.

[13] Yoshimura S., Knight-Jones T. (2012). Report on FAO/OIE global conference on foot and mouth disease control. *Journal of Veterinary Epidemiology*.

[14] Niedbalski W., Fitzner A., Bulenger K. (2019). Recent progress in vaccines against foot-and-mouth disease. *Medycyna Weterynaryjna*.

[15] Sangula A. K., Belsham G. J., Muwanika V. B. (2010). Evolutionary analysis of foot-and-mouth disease virus serotype SAT 1 isolates from East Africa suggests two independent introductions from southern africa. *BMC Evolutionary Biology*.

[16] Grubman M. J., Baxt B. (2004). Foot-and-mouth disease. *Clinical Microbiology Reviews*.

[17] Rweyemamu M. M., Booth J. C., Head M., Pay T. W. F. (1978). Microneutralization tests for serological typing and subtyping of foot-and-mouth disease virus strains. *Journal of Hygiene*.

[18] Hunter P. (1998). Vaccination as a means of control of foot-and-mouth disease in sub-saharan Africa. *Vaccine*.

[19] Paton D. J., Sumption K. J., Charleston B. (2009). Options for control of foot-and-mouth disease: knowledge, capability and policy. *Philosophical Transactions of the Royal Society B: Biological Sciences*.

[20] Paton D. J., Di Nardo A., Knowles N. J. (2021). The history of foot-and-mouth disease virus serotype C: the first known extinct serotype?. *Virus Evolution*.

[21] Rweyemamu M., Roeder P., Mackay D. (2008). Epidemiological patterns of foot-and-mouth disease worldwide. *Transboundary and Emerging Diseases*.

[22] Vosloo W., Bastos A. D. S., Sangare O., Hargreaves S. K., Thomson G. R. (2002). Review of the status and control of foot and mouth disease in sub-Saharan Africa. *Revue Scientifique et Technique de l’OIE*.

[23] Ahmed H. A., Salem S. A. H., Habashi A. R. (2012). Emergence of foot-and-mouth disease virus SAT 2 in Egypt during 2012. *Transboundary and Emerging Diseases*.

[24] Knowles N. J., Samuel A. R. (2003). Molecular epidemiology of foot-and-mouth disease virus. *Virus Research*.

[25] Bengis R. G., Thomson G. R., Hedger R. S., De Vos V., Pini A. (1986). Foot-and-mouth disease and the African buffalo (Syncerus caffer). 1. Carriers as a source of infection for cattle. *Onderstepoort Journal of Veterinary Research*.

[26] Maree F., Rweyemamu M., Kasanga C. (2014). Challenges and prospects for the control of foot- and-mouth disease: an African perspective. *Veterinary Medicine Research and Reports*.

[27] Vosloo W., Dwarka R. M., Bastos a. D. S., Esterhuysen J. J., Sahle M., Sangare O. Molecular epidemiological studies of foot-and-mouth disease virus in Sub-saharan Africa indicate the presence of large numbers of topotypes: implications for local and international control.

[28] Thomson G. R. (1995). Overview of foot and mouth disease in southern Africa. *Revue Scientifique et Technique de l’OIE*.

[29] Batho H. (2003). Report to the OIE following a request by the Southern African development community (SADC) for an emergency audit on foot-and-mouth disease (FMD) in Southern Africa. *OIE FMD Report SADC*.

[30] Jamal S. M., Shah S. I., Ali Q. (2014). Proper quality control of formulated foot-and-mouth disease vaccines in countries with prophylactic vaccination is necessary. *Transboundary and Emerging Diseases*.

[31] ElSayed E., Mossad W., Ali S., Shawky M. (2012). Studies on the duration of immunity induced in cattle after natural FMD infection and post vaccination with bivalent oil vaccine. *Veterinary World*.

[32] De Clercq B., Abatih E., Dal Pozzo F. (2018). Review of epidemiological risk models for foot-and-mouth disease: implications for prevention strategies with a focus on Africa. *PLoS One*.

[33] Bertram M. R., Bravo de Rueda C., Garabed R. (2018). Molecular epidemiology of foot-and-mouth disease virus in the context of transboundary animal movement in the far north region of Cameroon. *Frontiers in Veterinary Science*.

[34] Teye M. V., Sebunya T. K., Mpolokang Fana E. (2019). Foot-and-mouth disease in Southern Ghana: occurrence and molecular characterization of circulating viruses. *Tropical Animal Health and Production*.

[35] Babiuk S. (2018). Sample collection and transport. *Lumpy Skin Disease*.

[36] W. O. for A. H. (OIE) (2018). Foot and mouth disease (infection with foot and mouth disease virus). *Terrestrial Manual Online Access-OIE*.

[37] Jaffe A. (2014). *The Way Things Go*.

[38] Knowles N. J., Wadsworth J., Bachanek-Bankowska K., King D. P. (2016). VP1 sequencing protocol for foot and mouth disease virus molecular epidemiology. *Revue Scientifique et Technique de l’OIE*.

[39] Thompson J. D., Higgins D. G., Gibson T. J. (1994). Clustal W: improving the sensitivity of progressive multiple sequence alignment through sequence weighting, position-specific gap penalties and weight matrix choice. *Nucleic Acids Research*.

[40] Kumar S., Stecher G., Li M., Knyaz C., Tamura K., “Mega X. (2018). Mega X: molecular evolutionary genetics analysis across computing platforms. *Molecular Biology and Evolution*.

[41] Kimura M. (1980). A simple method for estimating evolutionary rates of base substitutions through comparative studies of nucleotide sequences. *Journal of Molecular Evolution*.

[42] Tamura K., Nei M. (1993). Estimation of the number of nucleotide substitutions in the control region of mitochondrial DNA in humans and chimpanzees. *Molecular Biology and Evolution*.

[43] Sangula A. K., Siegismund H. R., Belsham G. J., Balinda S. N., Masembe C., Muwanika V. B. (2011). Low diversity of foot-and-mouth disease serotype C virus in Kenya: evidence for probable vaccine strain re-introductions in the field. *Epidemiology and Infection*.

[44] Voslo G. K., Du Plessis B. J. A., Kloeck P. E. L. G. (2002). Foot and mouth disease: the experience of South Africa. *Revue Scientifique et Technique de l’OIE*.

[45] Meenowa D. (2020). Information Received on 14/09/2016 from Dr Deodass Meenowa, Assistant-director (livestock and veterinary services), division of veterinary services, Ministry of Agro-Industry and Food Security, REDUIT, Mauritius. https://www.oie.int/wahis_2/public/wahid.php/Reviewreport/Review?reportid=20849.

[46] Jamal S. M., Belsham G. J. (2013). Foot-and-mouth disease: past, present and future. *Veterinary Research*.

[47] Dn M., Kitala P. M. (2017). Epidemiological analysis of passive surveillance data on foot and mouth disease occurrence in Nakuru County, Kenya. *Journal of Dairy, Veterinary & Animal Research*.

[48] Sinkala Y. (2016). *FMD Zambia Information Received on 03/03/2016 from Dr Yona Sinkala*.

[49] Jori F., Vosloo W., Du Plessis B. J. A., Brahmbhatt D., Gummow B., Thomson G. R. (2009). A qualitative risk assessment of factors contributing to foot and mouth disease outbreaks in cattle along the western boundary of the Kruger National Park. *Revue Scientifique et Technique de l’OIE*.

[50] Scoones I., Bishi A., Mapitse N., Moerane R., Sibanda R., Wolmer W. (2010). Foot-and-mouth disease and market access: challenges for the beef industry in Southern Africa. *Pastoralism*.

[51] Derah N., Mokopasetso M. (2005). The control of foot and mouth disease in Botswana and Zimbabwe. *Tropicultura*.

[52] Vosloo W., Bastos A. D. S., Sahle M., Sangare O., Dwarka R. M. (2005). Virus topotypes and the role of wildlife in foot and mouth disease in Africa. *Conservation and Development Interventions at the Wildlifelivestock Interface: Implications for Wildlife, livestock and Human Health*.

[53] Paton D. J., Valarcher J. F., Bergmann I. E. (2005). Estudio de la selección de cepas vacunales contra la fiebre aftosa. *Revue Scientifique et Technique de l’OIE*.

[54] Dekker A., Chénard G., Stockhofe N., Eblé P. L. (2016). Proper timing of foot-and-mouth disease vaccination of piglets with maternally derived antibodies will maximize expected protection levels. *Frontiers in Veterinary Science*.

[55] Parida S. (2012). Vaccination against foot-and-mouth disease virus: strategies and effectiveness. *Expert Review of Vaccines*.

[56] Doel T. R. (2003). FMD vaccines. *Virus Research*.

[57] Sutmoller P., Barteling S. S., Olascoaga R. C., Sumption K. J. (2003). Control and eradication of foot-and-mouth disease. *Virus Research*.

